# Effects of climatic factors on rotavirus infection in Bangladesh: a time series analysis and future projections

**DOI:** 10.1080/16549716.2026.2707658

**Published:** 2026-07-30

**Authors:** Md Abu Sayeed, Syed M. Satter, Adriana Milazzo, Peng Bi, Keith Dear, Helen Marshall, Mahmudur Rahman, Md Rezanur Rahaman

**Affiliations:** a National Centre for Epidemiology and Population Health, The Australian National University, Canberra, Australia; b Programme for Emerging Infections, Infectious Diseases Division, International Centre for Diarrhoeal Disease Research, Dhaka, Bangladesh; c School of Public Health, Adelaide University, Adelaide, South Australia, Australia; d Adelaide Medical School and Robinson Research Institute, Adelaide University, Adelaide, South Australia, Australia; e Women’s and Children’s Research Centre, Women’s and Children’s Health Network, Adelaide, South Australia, Australia; f Institute of Epidemiology Disease Control and Research (IEDCR), Dhaka, Bangladesh

**Keywords:** Distributed lag non-linear model, hospitalisation, climate variability, paediatric diarrhoea, Bangladesh

## Abstract

**Background:**

Limited evidence exists concerning the association between climate factors and hospitalised rotavirus cases in Bangladesh.

**Objectives:**

To assess the association between temperature and hospitalised rotavirus cases among children aged < 5 years in Bangladesh.

**Methods:**

We obtained weekly hospital admissions data for rotavirus from seven surveillance hospitals and climate data from the Bangladesh Meteorological Department for August 2013–June 2017. We used a maximum temperature (t-max)–based distributed-lag non-linear model (DLNM) to estimate the association between temperature and hospitalised rotavirus cases, adjusting for seasonality using natural splines. We estimated relative risk (RR), attributable number, and attributable fraction (AF), and scenario-based exploratory projections for 2040 under different Representative Concentration Pathways (RCPs).

**Results:**

During the study period, 4,337 hospitalised rotavirus cases were reported among children <5 years. Countrywide, 1,781 (41%) cases were attributable to temperatures above the reference value (25.4°C), reflecting regional heterogeneity. Hospitalisation risk peaked at the 95th percentile of t-max (RR: 5.9; 95% CI: 3.6–9.7). Temperature–hospitalisation associations differed across divisions, with inverse or minimal associations observed in Dhaka, Rangpur, and Sylhet. Scenario-based exploratory projections suggested the highest risk at 0-week lag, with risk increasing again at week-4 lag above 41.7°C.

**Conclusions:**

Associations between t-max and rotavirus hospitalisation varied across Bangladesh, with higher temperatures associated with increased hospitalisation risk in some divisions but inverse or minimal associations in others, highlighting the importance of context-specific climate-sensitive interventions. Scenario-based projections beyond the observed temperature range should be interpreted cautiously, as these estimates represent exploratory extrapolations rather than forecasts of disease burden.

## Paper Context

- **Main findings:** The temperature–rotavirus relationship in Bangladesh exhibited pronounced spatial heterogeneity, with region-specific risk magnitudes and lag-response patterns.
- **Added knowledge:** It is a comprehensive national analysis using distributed lag non-linear modelling to disentangle temperature–rotavirus relationships while accounting for rainfall and humidity across multiple divisions.
- **Global health impact for policy and action:** Findings highlight the need for climate-sensitive rotavirus prevention strategies, including vaccination and strengthened water, sanitation, and hygiene interventions, particularly in vulnerable urban slum settings.

## Background

Globally, diarrhoea is the fourth leading cause of death among children aged < 5 years (hereafter, children < 5), with a mortality rate of 5.5 deaths per 100,000 children in this age group [1]. Worldwide, rotavirus infection is a major contributor, accounting for 35.7% of diarrhoeal deaths between 2017 and 2018 [2]. Rotavirus is seasonal, peaks in winter or early spring in temperate countries, and during dry seasons in tropical countries, with temperature, humidity, and rainfall influencing the risk of infection [3–5]. Earlier studies in tropical and subtropical settings have reported substantial heterogeneity in rotavirus seasonality, with peaks occurring during warmer or rainy seasons in some regions, and cooler or dry periods in others, particularly in densely populated urban environments [6–8]. Urbanisation, population density, Water, Sanitation and Hygiene (WASH) infrastructure, and healthcare access can influence exposure pathways and healthcare-seeking behaviours, thereby modifying temperature-associated patterns of rotavirus infection and hospitalisation [9]. Consequently, climate-related risk patterns may differ across administrative regions, indicating the importance of regionally stratified analyses to inform targeted public health interventions.

Bangladesh, as a tropical country, experiences heavy precipitation and tropical cyclones during monsoons. Inadequate sanitation and limited healthcare infrastructure increase the risk of rotavirus hospitalisation, which may be further intensified by rising temperatures, heavier rainfall and monsoon precipitation and more frequent flooding [10–13].

Despite long-standing rotavirus surveillance and pilot vaccination programmes coordinated by the International Centre for Diarrhoeal Disease Research, Bangladesh (icddr,b), sustained prevention and control of rotavirus have yet to be achieved in Bangladesh.

Government interventions including oral rehydration therapy, community-based awareness programmes, and improvements in child health services are hindered by poorer socioeconomic conditions, limited healthcare access, coupled with additional challenges posed by climate change [14]. Therefore, understanding the epidemiology between rotavirus infection and climatic conditions is important for informing preventive measures and reducing the burden of diarrhoeal diseases among young children in Bangladesh.

The aim of this study was to examine the relationship between ambient temperature and the occurrence of rotavirus cases in children <5 years of age in Bangladesh from 2013 to 2017, and to conduct scenario-based exploratory projections of future rotavirus risk under different Representative Concentration Pathways (RCP) scenarios. RCP-based temperature increments derived from the World Bank Climate Risk Country Profile for Bangladesh [15] were used for the exploratory projection analysis, as these estimates provided a consistent framework for scenario-based assessment of future temperature-related rotavirus risk. The year 2040 was selected as a near-term projection horizon to align with current public health planning and climate adaptation timelines in Bangladesh. Bangladesh-specific downscaled projections based on Shared Socioeconomic Pathways (SSPs) were not available for this analysis.

## Methods

### Rotavirus surveillance data

We used weekly hospitalisation data (August 2013 to June 2017) for rotavirus cases in children aged < 5 years reported from the childhood diarrhoea surveillance network consisting of seven tertiary hospitals, one in each of the seven administrative divisions of Bangladesh – Barisal, Chattogram, Dhaka, Khulna, Rajshahi, Rangpur, and Sylhet [16]. These referral hospitals are located in the most populous district of each administrative division, addressing both urban and rural catchment populations, providing broad geographic and socioeconomic coverage in Bangladesh.

Every fourth child < 5 years of age admitted to the designated surveillance hospitals with acute diarrhoea (≥3 loose stools per day with symptoms lasting for ≤7 days) [16] had a stool sample collected for rotavirus testing using enzyme-linked immunosorbent assay (ELISA) at the icddr,b laboratory, ensuring consistency in diagnosis. This systematic approach, based on admission order, has been validated in earlier surveillance evaluations to minimise selection bias and to ensure representative case profiles [14]. For administrative consistency, cases from the northern part of the Dhaka division were retained under Dhaka throughout the study period, although the Mymensingh division was officially established in 2015. Children from these areas continued to seek care at the existing surveillance hospital in Dhaka, so the catchment and representativeness remained unchanged, and data consistency across years was maintained. Formal seasonal comparisons of refusal to provide stool samples for rotavirus testing or differences in severity profiles between sampled and non-sampled children were not available, however, previous evaluations of the surveillance system did not identify systematic differences in age or clinical severity by sampling status or season [14].

### Meteorological data

We obtained weekly maximum (t-max), minimum (t-min), mean temperature (t-mean), relative humidity, and rainfall data for corresponding administrative divisions housing surveillance hospitals from the Bangladesh Meteorological Department. Daily meteorological observations from monitoring stations within each administrative division were aggregated into weekly averages to align temporally with weekly hospitalised rotavirus surveillance counts. Recordings were averaged to generate both countrywide and division-specific weekly climate data over the study period.

### Distributed lag non-linear model

We employed DLNMs using the *‘dlnm’* package in R [17] to assess the temporal effect of temperature over a maximum lag period of 4 weeks, both overall and by administrative division. Spearman’s correlation analysis and overdispersion checks were conducted, and the temperature cross-basis was constructed using a linear function for the exposure–response relationship and a polynomial function (degree 3) for the lag structure over a lag period of up to 4 weeks. Specifically, the exposure–response function was specified as linear, and the lag–response function was specified as a third-degree polynomial over a maximum lag of 4 weeks. No internal knots were used in the model for the exposure–response function due to the linear specification, and none were required for the lag dimension due to the use of a polynomial lag structure. We performed univariable analyses for t-mean, t-max, t-min, rainfall, and relative humidity, followed by multivariable regression models. In the multivariable models, we simultaneously included temperature indices along with rainfall and relative humidity (modelled using natural cubic splines with 3 degrees of freedom – DF) to account for the joint effects of multiple climate variables and to flexibly control for seasonality and long-term trends. For natural cubic spline terms (rainfall, relative humidity, and calendar time), knots were placed automatically by the *dlnm* package at equally spaced quantiles of the observed distributions according to the specified DF. We evaluated different combinations of degrees of freedom (3–6 DF) for meteorological variables and temporal trends, and selected the final model based on the lowest Akaike Information Criterion (AIC) (Suppl Table 1), with additional consideration of model parsimony and stability of effect estimates across alternative specifications. Where differences in AIC were minimal, simpler models were preferred to reduce the risk of overfitting. The t-max-based model was selected as the primary model to estimate lag-specific effects on hospitalised rotavirus cases, incorporating temperature cross-basis matrices and natural cubic splines for week (DF: 4 per year), relative humidity (DF: 3), and rainfall (DF: 3) to account for seasonality and long-term effects [18]. Temperature effects were modelled as continuous exposure–lag–response functions using absolute temperature values (°C). For interpretability, relative risks (RR) were additionally summarised at selected percentiles (50th, 75th, and 90th) of the observed t-max distribution; these summaries were derived directly from the fitted DLNM and did not involve re-specification of the model. Absolute temperature values were retained for all exposure–response curves, lag surfaces, and projection analyses. The multivariable model formulation was as follows:

$$\mathrm{Log}\left[E\left(y\right)\right]=\alpha +\beta 1\;\left(\mathrm{cb}.\mathrm{temperature}\right)+\mathrm{NS}\left(\mathrm{rain};\mathrm{DF}=3\right)+\mathrm{NS}\left(\mathrm{mean}\_\mathrm{rh};\mathrm{DF}=3\right)+\mathrm{NS}\left(\mathrm{Week},\;4\mathrm{DF}/\mathrm{year}\right)+\mathrm{Year}$$

Here, [E (y)] represents the expected weekly case count, cb.temperature represents the cross-basis matrix and β1 is the regression coefficient for different temperature indices, NS (rain; DF = 3) represents weekly rainfall modelled with a natural cubic spline (3 DF), NS (mean_rh; DF = 3) represents weekly mean relative humidity modelled with a natural cubic spline (3 DF), and NS (week; 4 DF/year) represents week modelled with a natural cubic spline (4 DF per year). Rainfall and relative humidity were included as time-varying covariates to adjust for short-term meteorological confounding, while long-term trends and seasonal variations were controlled for using a natural cubic spline for calendar week (4 DF per year). We examined model residuals using autocorrelation function (ACF) plots to assess serial correlation (Suppl Figure 1). After adjusting for seasonality and long-term trends, no substantial residual autocorrelation remained, suggesting that autoregressive terms were not required. The distribution of observed weekly t-max values was examined to assess data support across the exposure range, with sparse observations identified at the upper temperature tail (Suppl Figure 2).

Variance-to-mean ratios and residual diagnostics indicated overdispersion in weekly case counts; therefore, negative binomial regression models were used as the modelling framework. The dispersion parameter (θ) was estimated via maximum likelihood within the negative binomial model and was consistently greater than zero, confirming substantial overdispersion relative to a Poisson specification. The negative binomial model was additionally compared with quasi-Poisson models in sensitivity analyses. To assess regional variability, we evaluated spatial heterogeneity by examining effect modification by administrative division through inclusion of interaction terms between the temperature cross-basis and division, with models compared using likelihood ratio tests. For all DLNM-based analyses, relative risks (RR) were estimated with respect to a common reference temperature of 25.4°C, corresponding to the national average weekly mean temperature over the study period. This reference value was used for all national analyses. For division-specific exposure–response relationships, the same DLNM specification was used, but RRs were calculated against the division-specific average weekly mean temperature as the reference value from the observed data, rather than estimated within the model. This approach ensures that regional estimates reflect local temperature distributions rather than a common national exposure baseline. We evaluated alternative lag specifications, including higher-order polynomial forms, and observed similar overall exposure–lag patterns, supporting the robustness of the selected model.

### Lagged effects of different temperature indices on rotavirus infection and hospitalisation risk

We estimated the DLNM based on overall and division-specific RR and cumulative RR values for t-max, t-mean, and t-min. Rainfall and relative humidity were included in the models using natural cubic splines (3 DF each) to adjust for potential confounding and to account for seasonal patterns. Lagged effects were examined over lags 0–4 weeks, and results were presented graphically using exposure–response curves, lag–response slices, and three-dimensional plots.

### Effects of t-max variations on the risk of rotavirus infection

We calculated the overall and division-specific percentile values (50^th^, 75^th^, and 90^th^) of t-max, followed by their subsequent RRs and confidence intervals from the DLNM of rotavirus infection compared to the baseline values. For national analyses, RRs were estimated relative to the average countrywide weekly mean temperature, while for division-specific analyses, division-specific weekly mean temperatures were used as reference values [17]. These percentile-based estimates were used to summarise risks at commonly observed temperature ranges and to facilitate interpretation.

### Effects of t-max variations on the absolute risk of rotavirus infection and hospitalisation

We used ‘attrdl.R’ function in the R software package [19] to estimate the absolute risk (AR) of rotavirus infection associated with t-max. We calculated the backward attributable fraction (AF) and attributable number using risks estimated from the DLNM, referencing a counterfactual scenario of exposure indicated by the subsequent average value of the weekly t-mean for each temperature metrics for both overall and division level, while adjusting for rainfall and relative humidity as covariates. For countrywide analyses, attributable measures were estimated directly from the pooled national model using the full set of weekly observations. For division-specific analyses, attributable measures were estimated separately using the same DLNM specification but applying each division’s observed temperature distribution and division-specific reference temperature, thereby preserving regional climatic differences while maintaining a consistent modelling framework.

As attributable measures depend on both exposure–response function and the underlying exposure distribution, division-specific attributable numbers are not additive across regions. Consequently, the national attributable fraction represents a net summary of heterogeneous regional effects rather than a uniform temperature–risk relationship. Negative attributable fractions may arise when substantial proportions of cases occur below the selected reference temperature or where inverse exposure–response relationships are observed within parts of the temperature distribution; these estimates should be interpreted as distribution-dependent model summaries rather than direct causal effects.

To evaluate the sensitivity of AF and attributable number estimates to the choice of reference temperature, we conducted additional analyses using alternative baseline definitions. In addition to the primary analysis, we calculated AFs and attributable numbers using two alternative reference temperatures at both the countrywide and division level: the median observed t-max and the minimum-risk temperature (MRT) identified from the DLNM as the temperature associated with the lowest cumulative RR. Consistency of effect direction across alternative reference temperatures was assessed to confirm that attributable estimates were not driven by a single baseline choice (Suppl Table 2).

### Lagged effects of maximum temperature (t-max) on rotavirus infection and hospitalisation risk

Maximum temperature was selected as the primary exposure due to superior model fit based on AIC and exhibited greater parsimony and more stable exposure–lag patterns across divisions. In addition, maximum temperature was considered more biologically relevant for severe rotavirus outcomes, as peak daily heat may increase dehydration, alter virus survival, influence outdoor exposure, and care-seeking behaviour. These mechanisms are particularly relevant to severe cases requiring hospitalisation, making t-max conceptually a more appropriate metric than mean or minimum temperature.

### Scenario-based exploratory projections under future temperature conditions

We conducted scenario-based exploratory projections for the year 2040 using temperature increments derived from RCP scenarios 2.6, 4.5, 6.0, and 8.5 [15]. For each scenario, we incrementally added projected temperature increases (RCP 2.6: +1.1°C; RCP 4.5: +1.5°C; RCP 6.0: +1.2°C; RCP 8.5: +1.9°C) to the maximum observed t-max of 40.6°C. Using the DLNM cross-prediction function, we generated RR estimates across these extended temperature ranges, with the baseline set at 25.4°C. Lag-specific effects (0–4 weeks) were also calculated to capture short- and medium-term responses. These projections were implemented using the *crosspred* function in the R ‘dlnm’ package with the fitted model and are exploratory, intended to highlight the long-term public health relevance of our findings by informing preparedness planning, intervention timing, and geographic prioritisation in high-risk settings, with uncertainty intervals reflecting parametric uncertainty from the fitted DLNM rather than uncertainty in future climate projections. This approach applies uniform temperature increments as a scenario-based stress test rather than a forecast of day-to-day variability, allowing examination of potential risk responses under plausible warming magnitudes in the absence of high-resolution downscaled climate time series. These projections do not account for potential changes in the distribution of temperature under climate change, including shifts in variability, frequency, and intensity of extreme heat events, and should therefore be interpreted as scenario-based estimates of mean temperature displacement rather than full climate projections.

## Results

### Summary statistics

A total of 4,337 rotavirus cases were recorded from 2013 to 2017. Overall, the mean number of cases was 3 (standard deviation – SD: ±3) per week. Weekly average t-max was 30.9°C (SD: ±3.4), t-mean 25.4°C (SD: ±4.2) and t-min 21.1°C (SD: ±5.1). The mean weekly relative humidity was 79.5% (SD: ±7.0), and the average weekly rainfall was 6.2 mm (SD: ±11.0). Division-specific variations in temperature, humidity, and rainfall are presented in Table 1.

**Table 1.** Descriptive statistics of weekly rotavirus cases, temperature, relative humidity, and rainfall from 2013 to 2017 in Bangladesh.

Administrative level	N	% of total cases	Count – M ± SD (min–max)	Maximum temperature (°C) – M ± SD (min–max)	Mean temperature (°C) – M ± SD (min–max)	Minimum temperature (°C) – M ± SD (min–max)	Relative humidity (%) – M ± SD (min–max)	Rainfall (mm) – M ± SD (min–max)
Overall (Countrywide)	4337		3 ± 3.7 (0–29)	30.9 ± 3.4 (20.4–40.6)	25.4 ± 4.2 (14.4–33.4)	21.1 ± 5.1 (8.9–28.2)	79.5 ± 7.0 (49.2–95.0)	6.2 ± 11.0 (0–122.1)
**Administrative division**								
Barisal	966	22.3	4 ± 2.7 (0–14)	31.0 ± 3.0 (22.1–38.3)	25.6 ± 4.2 (16.1–31.5)	21.7 ± 5.2 (9.8–27.9)	83.3 ± 5.6 (66.8–95.0)	5.6 ± 9.7 (0–75.2)
Chattogram	243	5.6	1 ± 1.3 (0–7)	30.5 ± 2.5 (23.7–36.1)	26.2 ± 3.2 (18.6–31.6)	22.4 ± 4.1 (13.5–28.3)	77.7 ± 7.4 (49.2–91.8)	8.2 ± 16.5 (0–122.1)
Dhaka	648	14.9	3 ± 3.3 (0–17)	30.0 ± 3.1 (21.5–36.6)	24.9 ± 4.2 (15.3–30.2)	20.9 ± 5.2 (9.8–27.4)	81.7 ± 4.9 (66.5–91.7)	5.5 ± 7.7 0–31.5)
Khulna	348	8.0	1 ± 2.0 (0–9)	32.0 ± 3.7 (21.8–40.6)	25.9 ± 4.7 (15.8–33.4)	21.0 ± 5.8 (8.9–29.9)	77.2 ± 6.4 (57.1–91.2)	4.1 ± 6.6 (0–38.1)
Rajshahi	1184	27.3	5 ± 6.3 (0–29)	31.7 ± 4.2 (21.0–40.2)	25.3 ± 4.9 (15.0–33.0)	20.6 ± 5.8 (8.9–27.5)	79.8 ± 7.2 (54.7–92.0)	3.5 ± 5.3 (0–29.5)
Rangpur	284	6.6	1 ± 2.9 (0–15)	29.8 ± 3.6 (20.4–36.7)	24.7 ± 4.5 (14.4–30.7)	20.2 ± 5.2 (9.6–27.4)	78.8 ± 6.7 54.4–90.7)	4.9 ± 8.6 (0–52.4)
Sylhet	664	15.3	3 ± 2.9 ( 0–12)	30.8 ± 2.8 (22.4–38.3)	24.9 ± 3.6 (16.6–30.6)	20.9 ± 4.4 (11.5–26.7)	78.0 ± 8.0 (53.1–94.0)	11.4 ± 15.2 (0–73.4)

* % indicating division contributing for countrywide case count * M ± SD – mean ± standard deviation.

Division-specific: Rajshahi reported the highest average number of weekly rotavirus cases of 5 (SD: ±6.3), while Chattogram had the lowest average of 1 (SD: ±1.3). Khulna recorded the highest average weekly maximum temperature of 32.0°C (SD: ±3.7), while Rangpur had the lowest at 29.8°C (SD: ±3.6). Barisal reported the highest weekly average relative humidity of 83.3% (SD: ±5.6). Sylhet recorded the highest average weekly rainfall of 11.4 mm (SD: ±15.2), and Rajshahi recorded the lowest average rainfall of 3.5 mm (SD: ±5.3) (Table 1).

### Overall impact of lag based different temperature indices on rotavirus infection and hospitalisation

**Figure 1.**
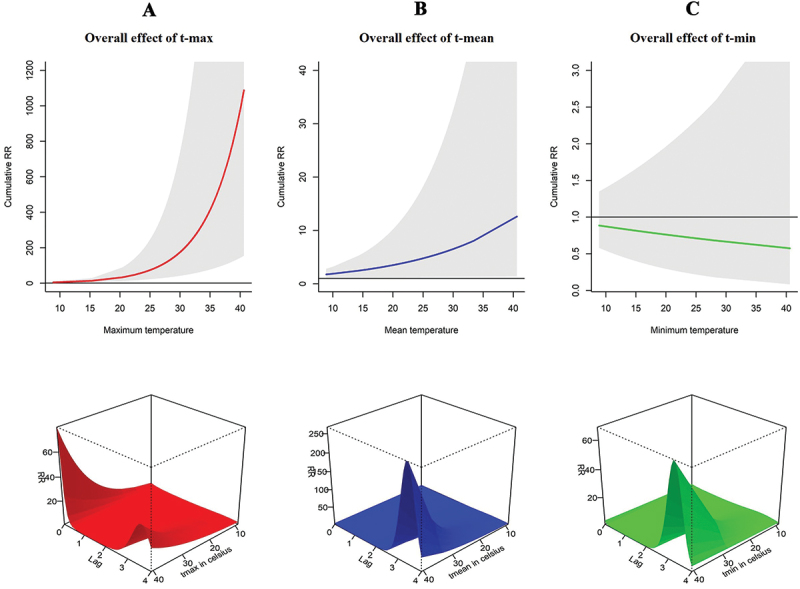
Overall cumulative plot and three-dimensional plot of risk in different temperatures. Column A: maximum temperature (t-max); column B: mean temperature (t-mean); column C: minimum temperature (t-min). All cumulative RRs are expressed relative to a reference temperature of 25.4°C, corresponding to the national average value of the weekly t-mean during the study period.

In the t-max–based DLNM, the overall cumulative RR increased with rising temperature and reached its highest values at the upper extreme of the observed exposure range (40.6°C). However, estimates at this upper temperature tail were associated with very wide confidence intervals, reflecting sparse observations and reduced model stability, and should be interpreted cautiously (Figure 1(A)). In the t-mean–based model, cumulative risk increased more gradually, with a maximum cumulative RR of 12.6 (95% CI: 1.4–111.3) at 40.6°C (Figure 1(B)). In contrast, the t-min–based model showed a decreasing association with increasing temperature (Figure 1(C)). The lag–temperature–risk association was further illustrated in a contour plot (Suppl Figure 3), showing the highest RR temperatures at 35°C, primarily at lag 0-week and 4-week, while risks remained low at lower temperatures across all lag periods.

Division-specific: exposure–response curves demonstrated substantial heterogeneity in both the magnitude and direction of temperature effects across administrative divisions; the effects of different temperature index–based models on rotavirus infection are shown in Suppl Figure 4. Formal interaction testing confirmed statistically significant effect modification by administrative division (likelihood ratio test, *p* < 0.001), indicating that temperature–rotavirus associations differed across divisions.

### Effects of t-max percentiles on the risk of rotavirus infection and hospitalisation

Overall, we observed an increase in the risk of rotavirus infection (RR: 2.8; 95% CI: 2.1–3.8), at t-max 50^th^ percentile, followed by a further escalation at the 75^th^ percentile (RR: 3.9; 95% CI: 2.7–5.7) and 90^th^ percentile (RR: 5.9; 95% CI: 3.6–9.7) (Table 2).

**Table 2.** Relative risk (RR) of rotavirus cases at different percentiles of t-max at overall and division level.

Administrative level	Ref temperature* (°C)	Category	Temperature (°C)	RR	95% CI
Overall (Countrywide)	25.4	50^th^ percentile	31.5	2.8	2.1–3.8
		75^th^ percentile	33.3	3.9	2.7–5.7
		95^th^ percentile	35.7	5.9	3.6–9.7
**Administrative division**					
Barisal	25.6	50^th^ percentile	31.7	2.5	1.3–4.8
		75^th^ percentile	32.9	3.0	1.4–6.6
		90^th^ percentile	35.3	4.4	1.5–12.2
Chattogram	26.2	50^th^ percentile	30.9	1.6	0.5–4.9
		75^th^ percentile	32.3	1.8	0.4–8.0
		90^th^ percentile	33.9	2.1	0.3–13.9
Dhaka	24.9	50^th^ percentile	30.8	0.7	0.3–1.4
		75^th^ percentile	32.5	0.6	0.2–1.6
		90^th^ percentile	33.9	0.5	0.1–1.7
Khulna	25.9	50^th^ percentile	32.7	2.8	0.8–9.2
		75^th^ percentile	34.4	3.6	0.7–16.1
		90^th^ percentile	37.3	5.5	0.7–41.6
Rajshahi	25.3	50^th^ percentile	32.8	1.5	0.8–2.7
		75^th^ percentile	34.8	1.7	0.7–3.6
		90^th^ percentile	36.8	1.8	0.7–4.8
Rangpur	24.7	50^th^ percentile	30.8	0.8	0.2–2.3
		75^th^ percentile	32.5	0.7	0.2–2.9
		90^th^ percentile	34.1	0.7	0.1–3.7
Sylhet	24.9	50^th^ percentile	31.2	0.9	0.4–1.7
		75^th^ percentile	33.1	0.8	0.3–2.1
		90^th^ percentile	34.7	0.8	0.3–2.4

*Ref temperature: average value of the weekly t-mean.

Division-specific ― Khulna had the highest RR of 5.5 (95% CI: 0.7–41.6) at the 90^th^ percentile of t-max, relative to the reference temperature (25.9°C). Barisal also showed a markedly elevated RR of 4.4 (95% CI: 1.5–12.2) at the 90th percentile (35.3°C), although not as high as that observed in Khulna. In contrast, Sylhet exhibited minimal changes in RR compared with the reference temperature (24.9°C) (Table 2).

Differences between percentile-based estimates (Table 2) and cumulative exposure–response curves (Suppl Figure 4) arise because percentile-based RRs estimate risk at specific temperature levels, whereas cumulative curves summarise the overall effect across both temperature and lag periods. Accordingly, the direction and magnitude of effects may differ between these two approaches.

### Attributable risk of rotavirus infection and hospitalisation due to t-max

Overall, we estimated 1781/4337 (41.1%) cases were attributable to temperatures higher than 25.4°C during the study period (Table 3). Division-specific analyses demonstrated substantial heterogeneity in temperature-attributable burden. Barisal showed the highest attributable fraction (50.0%), followed by Khulna (30.2%) and Chattogram (22.8%), whereas Dhaka (−32.7%), Rangpur (−3.7%), and Sylhet (−8.3%) exhibited negative attributable fractions (Table 3). Exposure–response heterogeneity across divisions is shown in Suppl Figure 5. Results of sensitivity analyses using alternative reference temperatures are presented in Suppl Table 2.

**Table 3.** Attributable risk (AR) (backward) and attributable fraction (AF) of rotavirus cases at overall and division level.

Administrative level	Ref temperature* (°C)	Total attributable number	Total attributable fraction (%)
Overall (countrywide)	25.4	1781	41.1
Division			
Barisal	25.6	483	50.0
Chattogram	26.2	55	22.8
Dhaka	24.9	−212	−32.7
Khulna	25.9	105	30.2
Rajshahi	25.3	161	13.6
Rangpur	24.7	−10	−3.7
Sylhet	24.9	−54	−8.3

*Ref temperature: average value of the weekly t-mean.

### Lag specific effects of t-max on the risk of rotavirus infection and hospitalisation

**Figure 2.**
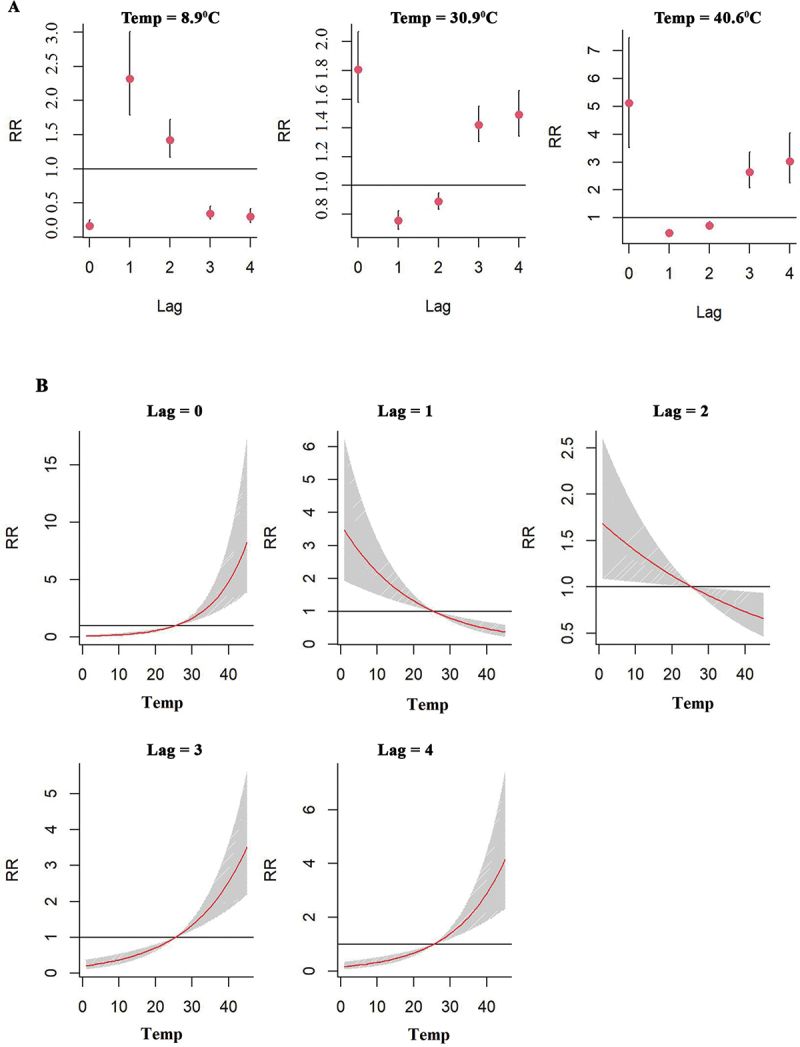
The estimated effects of temperatures on rotavirus cases at selected lags. 2/A: RR vs lag at different temperature; 2/B: RR vs temperature at different lag where the shaded areas represent 95% confidence intervals. Column A: ref temperatures are 8.9°C (the lowest countrywide temperature during the study period), 30.9°C (the mean temperature during the study period) and 40.6°C (the maximum temperature during the study period).

At 8.9 °C, the risk of infection and hospitalisation was lowest at lag 0-week (RR: 0.2; 95% CI: 0.1–0.3) and highest at lag 1-week (RR: 2.3; 95% CI: 1.6–3.4). At 30.9 °C, the risk of infection and hospitalisation was highest at lag 0-week (RR: 1.8; 95% CI: 1.5–2.2) then at lag 4 (RR: 1.5; 95% CI: 1.3–1.8). At 40.6 °C, the risk of infection and hospitalisation was highest at lag 0-week (RR: 5.1; 95% CI: 2.9–9.1) followed by lag 4 (RR: 3.0; 95% CI: 1.9–4.7) (Figure 2(A and B)).

### Projected t-max and lagged effects on future risk of rotavirus infection and hospitalisation

**Figure 3.**
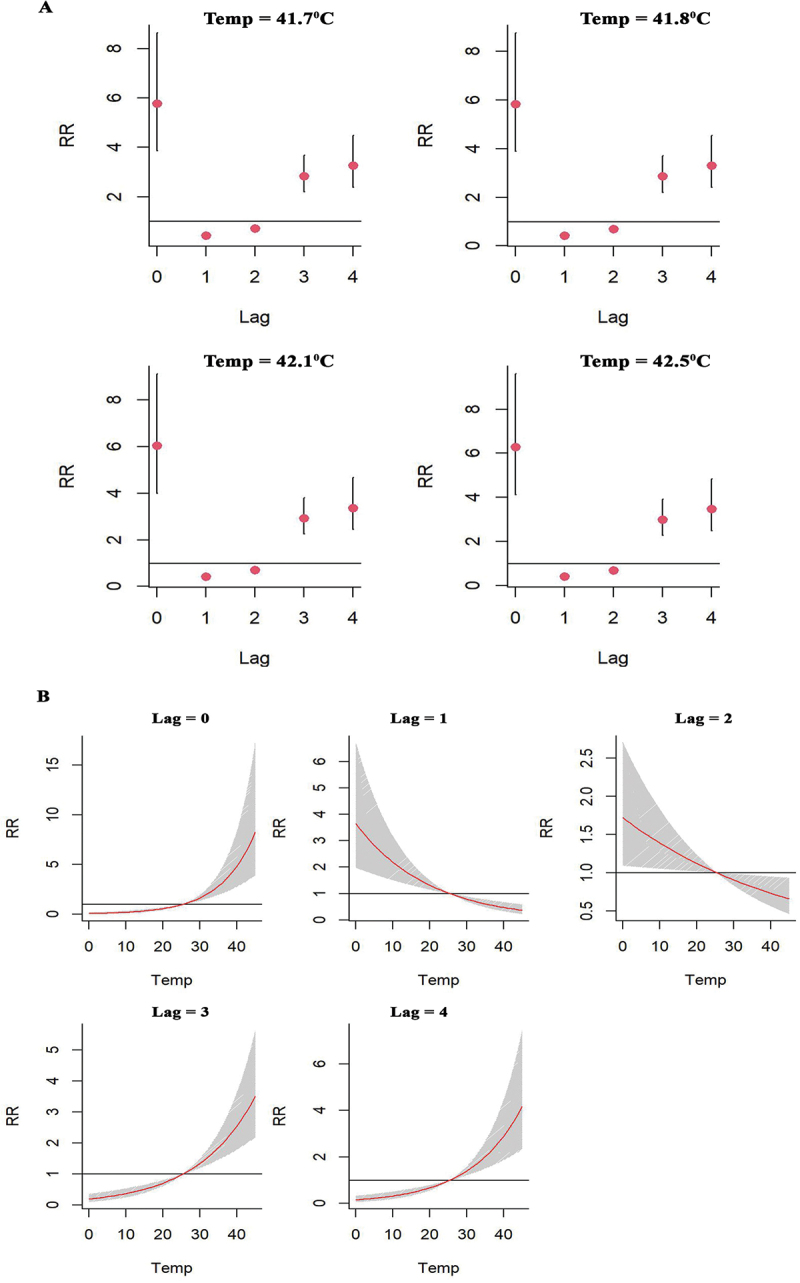
The estimated projected effects of temperatures on rotavirus cases at different lag. 3/A: RR vs lag at different temperature; 3/B: RR vs temperature at different lag where the shaded areas represent 95% confidence intervals.

Overall, at lag 1-week, the RR decreased from 3.6 to 0.4 as temperatures increased from 0°C to 45°C, a similar pattern was observed at lag 2-week, where RR values declined from 1.7 to 0.6, indicating a consistent response to temperature changes. However, the RR increased with increasing temperatures (8.9°C to 42.5°C) ranging from 0.2 to 3.5 at lag 3-week and from 0.1 to 4.2 at lag 4-week. At different temperatures of 41.7°C, 42.1°C, 41.8°C, and 42.5°C, the risk of infection and hospitalisation gradually decreased from lag 0-week to lag 1-week, remained relatively stable until lag 2-week, and then steadily increased, reaching its maximum at lag 4-week (Figure 3). At lag 0-week, the highest RR values were 5.7 (95% CI: 3.1–10.6), 5.8 (95% CI: 3.1–10.8), 6.0 (95% CI: 3.2–11.3), and 6.3 (95% CI: 3.3–12.0) corresponding to temperatures of 41.7°C, 41.8°C, 42.1°C, and 42.5°C, respectively. At lag week-4 the estimated RR values were 3.2 (95% CI: 2.0–5.3), 3.3 (95% CI: 2.0–5.4), 3.4 (95% CI: 2.1–5.5), and 3.5 (95% CI: 2.1–5.8) for the same temperatures. At lag 0-week, the projections indicated the highest RR values at extreme temperatures, whereas at lag 3-weeks and 4 the RR increased again under sustained higher temperatures (Figure 3(A and B)). These estimates extend beyond the observed temperature range; they should be interpreted as exploratory projections rather than validated forecasts of future disease burden.

## Discussion

Water, sanitation, and hygiene (WASH) and temperature variability may influence rotavirus-associated disease patterns, particularly in low- and middle-income countries (LMICs) like Bangladesh, necessitating investigation into the climate-rotavirus relationship for effective prevention and control [20]. Hospital-based surveillance system in Bangladesh captured severe pediatric diarrhoea cases across tertiary hospitals, with the network design ensuring broad geographic coverage and representation of both urban and rural catchment populations. Standardised diagnostic protocols and sampling procedures across sites further strengthened consistency and minimised systematic bias, providing a robust foundation for assessing climatic influences on hospitalised rotavirus cases and severe diarrhoeal disease patterns. Importantly, our findings indicate that temperature–rotavirus associations are not spatially uniform across Bangladesh, highlighting the need to interpret national summaries with caution due to regional heterogeneity.

In this study, we observed a strong association between t-max and hospitalised rotavirus cases across different lag periods, with notable variation between administrative divisions. The non-linear relationship emphasises the importance of using t-max-based modelling approaches and is consistent with a previous study [4] which found reporting increased risk of rotavirus infection at higher temperatures (mean temperature above 29.0°C). Our study consistently linked higher temperatures to an increased risk of hospitalised rotavirus cases, particularly at higher percentiles, reflecting a combination of environmental persistence of rotavirus, dehydration severity, healthcare-seeking behaviour, and other temperature-sensitive exposure pathways [21]. Increased temperatures may alter behavioural patterns, including outdoor activities, water contact, and healthcare-seeking behaviour among children, which may contribute to higher observed rates of rotavirus-associated hospitalisation through increased exposure opportunities and changes in healthcare utilisation patterns [22]. Additionally, heat stress and dehydration may exacerbate disease severity, increasing the likelihood that children with rotavirus infection require hospital care [23]. The estimated risk of the mean of t-max was greater compared to the minimum temperature (8.9°C), contradicting higher infection rates in temperate locations at cooler and drier seasons as reported by an earlier study [24]. Such differences likely reflect contextual variation and differences in model specification, including adjustment for rainfall, humidity, and delayed effects, which are not consistently addressed in earlier studies. Although factors such as dehydration, heat stress, and temperature-related changes in caregiving practices (e.g. altered fluid provision, feeding patterns, or care-seeking behaviour) may influence disease severity and healthcare utilisation rather than transmission risk, these mechanisms cannot be directly assessed using hospital-based surveillance data.

The national attributable fraction was estimated directly using the DLNM-based attribution framework applied to the pooled national time series. Division-specific attributable fractions were estimated separately to illustrate regional heterogeneity rather than derive the national estimate. Negative values reflect a predominance of cases below the reference temperature and should not be interpreted as direct causal effects, but rather as model-based estimated conditional on the observed exposure distribution and selected reference temperature.

Dhaka, the capital of Bangladesh, is a highly populated city where 35% of people live below the poverty line with more than 4,000 slum areas [25]. Rotavirus cases in Dhaka were predominantly reported at temperatures below the weekly mean (24.9°C) during dry seasons [26], aligning with studies from Nepal and the USA showing higher rotavirus risk during dry seasons [24,27]. This likely reflects the higher concentration of hospitalised rotavirus cases during cooler months in Dhaka’s overcrowded slum settings, where poor ventilation, close contact, and limited sanitation may amplify winter transmission and healthcare presentation patterns [4]. The negative attributable estimates observed in Dhaka suggest a different temperature–hospitalisation pattern compared with other divisions, although the underlying reasons remain unclear. Similar temperature–hospitalisation patterns were also observed in Rangpur and Sylhet, which experience cooler and wetter climates compared to other divisions. Division-specific attributable estimates revealed substantial regional contrasts, with Barisal showing the highest attributable fraction (50.0%), likely due to high humidity and rainfall potentially favouring viral persistence [21], while Rangpur and Sylhet, like Dhaka, showed negative estimates consistent with cooler-season clustering of cases.

An earlier study in Nepal documented seasonality (e.g. winter) associated with changes in sanitary habits among children living in overcrowded conditions to protect them from the cold [20], which aligns with this study findings in Dhaka, where 42.3% of slum households exceed five members and face overcrowding, particularly during winter [28]. These findings are consistent with observed temperature–hospitalisation patterns in some districts, including Dhaka. Moreover, the minimal changes in RR observed in Rangpur and Sylhet may reflect their cooler and wetter climates, where rotavirus-associated hospitalisation patterns remain relatively stable across temperature ranges. Conversely, wide confidence interval for Khulna at the 90th percentile likely reflects the strong but variable impact of extreme heat in this coastal division. However, fluctuating humidity and frequent saline water intrusion may interact with temperature and environmental exposure pathways to influence observed hospitalisation patterns, although this interpretation should be considered exploratory and warrants further investigation.

Recognising seasonal case patterns is important for optimising public health interventions. An earlier study in Dhaka found that high river levels and extreme heat may increase rotavirus hospitalisations, possibly through flooding-related contamination and increased water contact [4]. This suggests that the transition from the typical cool-season peaks may reflect complex interactions between temperature, humidity, flooding, and social behaviours, warranting cautious interpretation and further validation.

This study revealed that while lag 1–3 weeks showed modest effects, the risk of hospitalised rotavirus cases noticeably increased at lag 4-week, suggesting delayed environmental, behavioural, or healthcare-related responses associated with sustained temperature exposure or other temporal factors. At lag 0-week, projections consistently showed the highest immediate risk at extreme temperatures, whereas increasing RR values at lag 3–4 weeks indicate that the temperature–hospitalisation association may persist beyond the initial exposure period. The projected temperature–rotavirus associations shown in this study extend beyond the maximum observed temperature during the study period (40.6°C) and should therefore be interpreted with caution. Relative risk estimates at these extreme temperatures are subject to increased uncertainty due to limited empirical support, with wider confidence intervals reflecting reduced model stability outside the observed exposure range, and the projections are intended as illustrative scenario analyses rather than predictive forecasts. The large cumulative RR estimates at the upper temperature range likely reflect sparse observations, reduced model stability, and extrapolation beyond well-supported exposure values. Although the highest estimated RR occurred at the upper extreme of the observed temperature range (40.6°C), these estimates should be interpreted cautiously given the limited data support and instability of model estimates at extreme temperatures. The risk at lag 0-week for the maximum temperature values of t-max was greatest, while at the minimum temperature values of t-max, the risk was notably lower. These projections assume temporal stationarity of temperature–health relationships and do not account for potential behavioural, infrastructural, or healthcare adaptations. Overall, elevated temperatures were associated with changes in hospitalised rotavirus patterns, with distinct lag-response relationships observed across temperature ranges. In contrast, minimum temperature showed an opposite pattern compared to mean and maximum temperatures, possibly reflecting greater viral stability and indoor crowding during colder conditions.

Future studies should investigate how temperature influences rotavirus-associated hospitalisation dynamics, particularly because rotavirus survival may be greater at lower temperatures [24,29]. The associations between behavioural adaptation, healthcare access, WASH conditions, public health interventions and climate–disease relationships under changing temperature conditions should be evaluated [30,31]. Strengthening sanitation, healthcare and WASH infrastructure will be essential to mitigate climate related impacts on childhood diarrhoeal disease like rotavirus, given the substantial contribution of bacterial enteropathogens reported among children with diarrhoea in LMICs [32]. Furthermore, focusing on immunisation of children against rotavirus is crucial. Bangladesh has shown significant progress in WASH activities as a disease control approach [33] but vaccination for rotavirus is yet to be included in the national immunisation programme [34]. In addition to vaccination, further strengthening WASH interventions, improving nutritional support for children, and ensuring timely access to oral rehydration therapy remain essential complementary strategies to reduce disease burden in the face of climate variability. Inclusion of country-level rotavirus vaccination programmes have shown to be effective in reducing diarrhoea prevalence and mortality in India [35], Thailand [36], and Australia [37]. Moreover, many countries like the USA, Austria, Belgium, Latvia, and the UK have recorded herd immunity as a result of rotavirus vaccination [38]. Rotavirus immunisation initiatives can reduce the economic costs associated with managing infection. Our findings suggest the potential value of estimating the cost-effectiveness of geographically and seasonally targeted rotavirus vaccination campaigns, particularly in densely populated urban settings such as Dhaka.

This study benefits from multi-year, multi-site surveillance with standardised diagnostics and a DLNM that adjusts for seasonality, rainfall, and humidity. However, the hospital-based data may have captured only severe pediatric diarrhoea cases limiting the representativeness for less severe community-level infections. Division-level analyses are ecological in nature and subject to ecological fallacy, meaning that associations observed at the group or population level may not necessarily reflect individual-level risks or exposures. In addition, differences in referral patterns, healthcare access, tertiary hospital catchment populations, and healthcare-seeking behaviour across administrative divisions may have contributed to regional variation in observed hospitalisations. The systematic nature of the hospital sampling may influence representativeness of the study population. While site-level baseline data were not available for all hospitals, previous evaluations of the national surveillance system indicated no substantial differences in age or symptom severity between tested and untested children. Admission practices followed clinical criteria and did not vary systematically by season, reducing the risk of bias in temperature-related associations. Despite a relatively modest subset of laboratory-confirmed rotavirus cases, the multi-year, multi-site surveillance design provided sufficient power for national and divisional analyses.

The seasonal differences in refusal to provide stool samples or severity profiles between sampled and non-sampled children were not assessed. This may have introduced residual sampling bias related to admission patterns or care-seeking behaviour during extreme weather. The socioeconomic and infrastructure factors such as WASH access, crowding, healthcare access, or flooding, which co-vary seasonally with temperature, were also not measured; therefore, causal inference is limited, and some residual confounding may remain. In addition, exposure assignment relied on division-level meteorological data, which may not fully capture microclimatic variation or individual-level exposure, introducing potential ecological misclassification. Moreover, wide confidence intervals at higher temperature percentiles reflect sparse data and reduced model stability at extreme temperatures, limiting the precision of subnational attributable estimates. Therefore, the extreme cumulative RRs at the highest temperatures should not be interpreted as precise effect magnitudes, but rather as indicators of increased uncertainty arising from limited observations at the highest temperatures.

For projections, we used RCP-based temperature increments rather than SSP-linked pathways, so the projections are illustrative and do not include socioeconomic trajectories. In addition, projections implicitly assume temporal stationarity of temperature–health relationships and do not account for potential behavioural or infrastructural adaptation over time. We did not perform rotavirus strain-level genotyping and therefore cannot assess whether temperature effects differed by circulating genotypes or strain replacement over time. In addition, although standardised ELISA protocols were used across all surveillance sites, independent verification of uniform testing intensity across hospitals and seasons was not available. The findings also suggest higher risks at warmer temperatures. However, because previous studies in tropical and subtropical regions have reported winter peaks, these results should be interpretated with caution. As this study included hospitalised cases only, milder community infections were likely under-represented. Accordingly, the observed temperature–rotavirus associations primarily reflect patterns associated with severe diarrhoeal disease requiring hospitalisation rather than broader community transmission dynamics. Future work should consider division-level WASH and socioeconomic indicators, and mixed-effects or causal time-series approaches to further isolate temperature effects.

## Conclusions

Associations between ambient temperature and hospitalised rotavirus cases varied substantially across administrative divisions and lag periods in Bangladesh, with higher temperatures associated with increased risk in some divisions but inverse or minimal associations in others. These findings indicate that the pooled national estimate reflects heterogeneous regional patterns rather than a uniform heat-related increase in rotavirus risk across Bangladesh. The findings also highlight the importance of context-specific, climate-sensitive risk assessments to better understand rotavirus-related disease patterns in Bangladesh. Given the substantial disease burden and seasonal pressures on the health system, especially in rural areas, strengthening preventive strategies remains essential. These findings support further cost-effectiveness analysis of rotavirus vaccination alongside WASH and community-based interventions as part of broader climate-sensitive prevention strategies. Overall, our findings may help inform the development and prioritisation of future climate-sensitive prevention strategies tailored to regional disease patterns in Bangladesh. Further intervention and implementation studies are needed to assess their effectiveness under future climate changes. However, projected RR estimates beyond the observed temperature range should be interpreted cautiously, as they represent exploratory extrapolations under scenario-based warming assumptions.

## Supplementary Material

Revised Supplementary file Cleaned Upload.docx

## Data Availability

The rotavirus surveillance data used in this manuscript may be made available upon reasonable request to the corresponding author.
