# Supplementary material for: Effects of climatic factors on rotavirus infection in Bangladesh: a time series analysis and future projections
Source: Glob Health Action. 2026 Jul 30;19(1):2707658. doi: 10.1080/16549716.2026.2707658 (PMC13425533; doi:10.1080/16549716.2026.2707658)
Supplement: Revised Supplementary file Cleaned Upload.docx [file ZGHA_A_2707658_SM3017.docx]

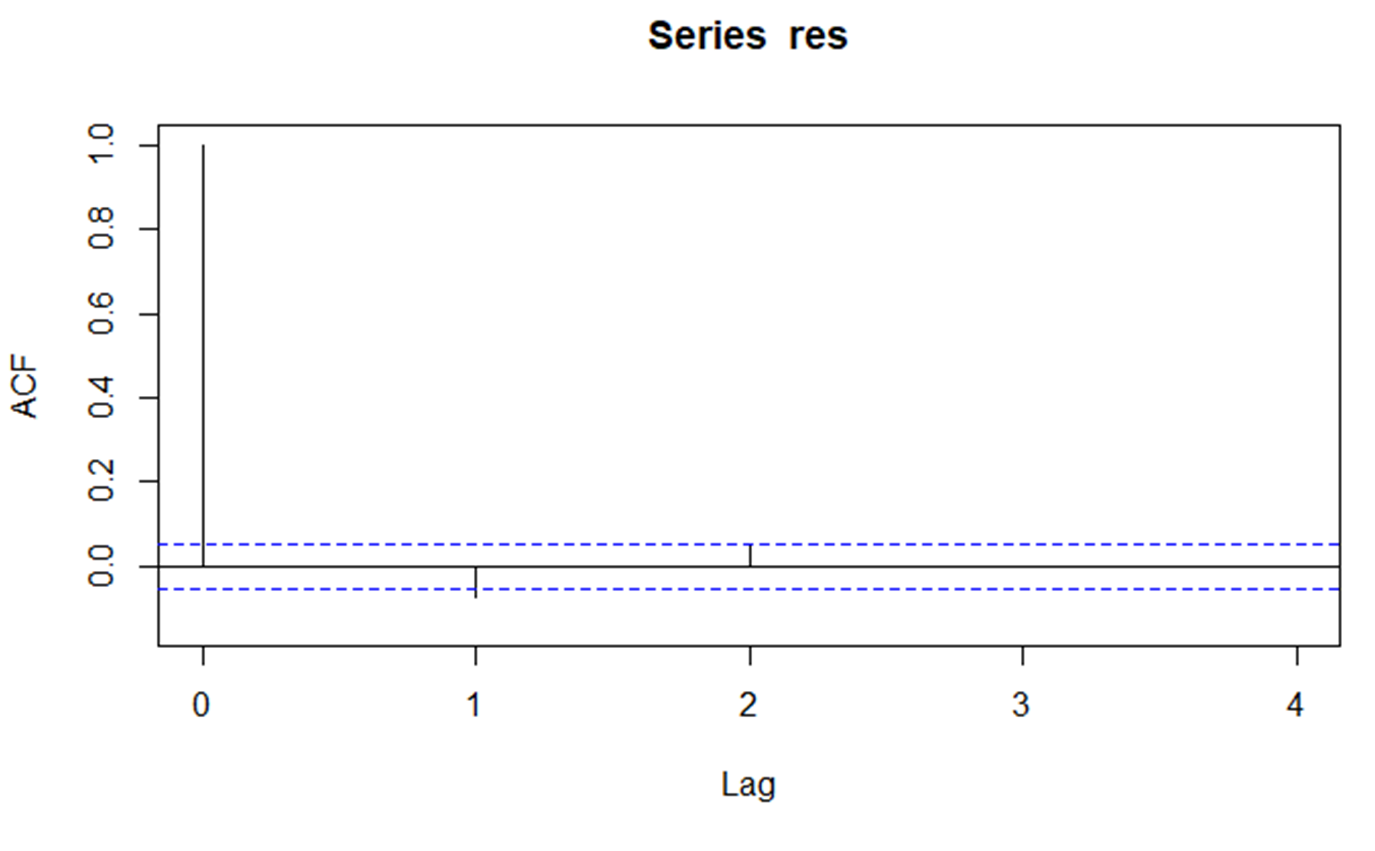

Suppl Figure 1: Autocorrelation function (ACF) plot of model residuals

Suppl Table 1: Model selection based on AIC values

| Temperature | AIC value | Degrees of freedom for relative humidity and rainfall | Degrees of freedom for time (per year) |
| --- | --- | --- | --- |
| Maximum temperature  (t-max) | 6421.6 | 3 | 3 |
|  | 6429.3 | 4 | 4 |
|  | 6443.3 | 5 | 5 |
|  | 6448.4 | 6 | 6 |
|  | 6347.4 | 3 | 4 |
| Mean temperature  (t-mean) | 6376.3 | 3 | 3 |
|  | 6387.9 | 4 | 4 |
|  | 6396.2 | 5 | 5 |
|  | 6405.8 | 6 | 6 |
|  | 6353.0 | 3 | 4 |
| Minimum temperature  (t-min) | 6382.1 | 3 | 3 |
|  | 6399.8 | 4 | 4 |
|  | 6411.8 | 5 | 5 |
|  | 6426.6 | 6 | 6 |
|  | 6351.4 | 3 | 4 |


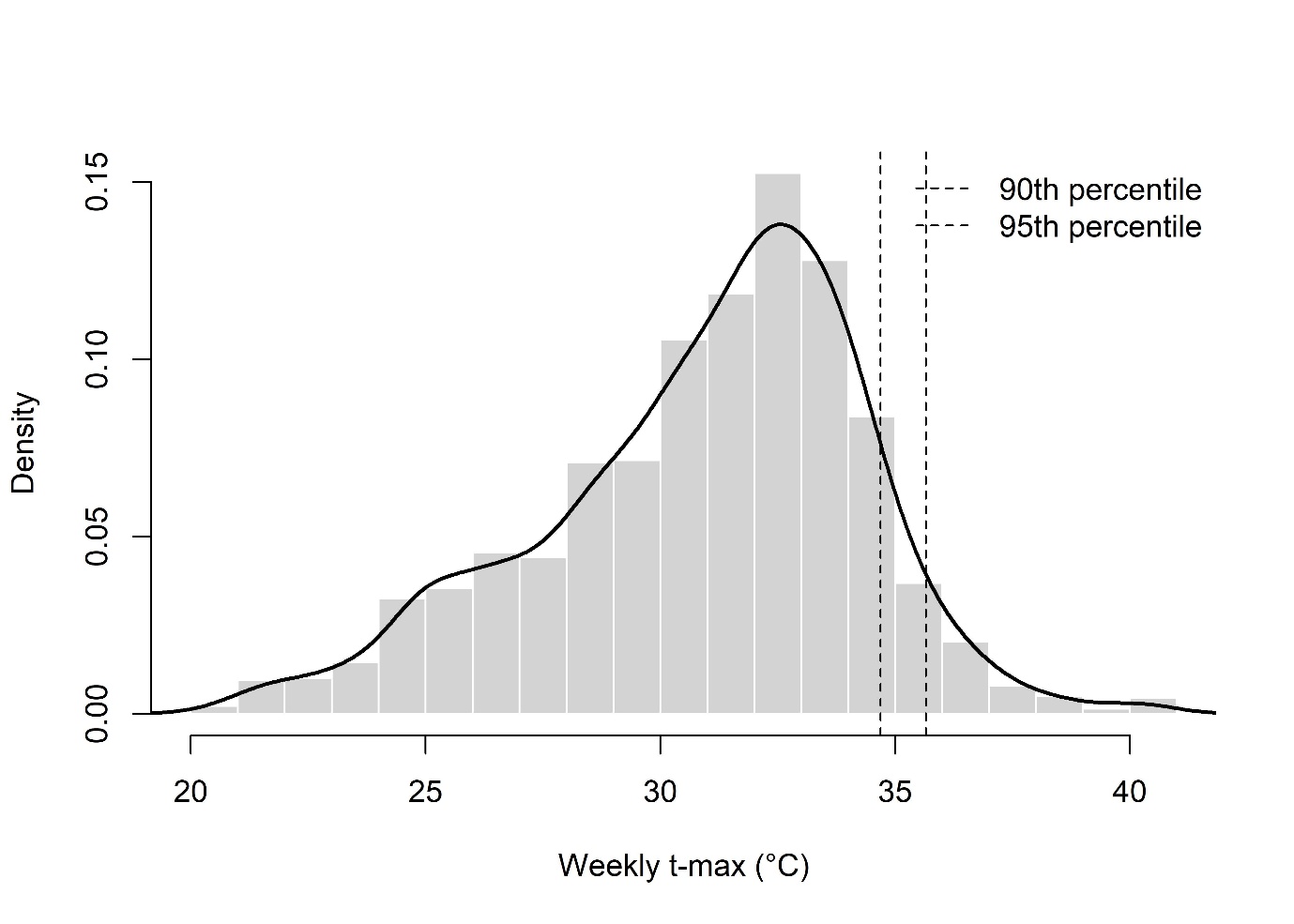


Suppl Figure 2: Distribution of observed weekly t-max values across the study period. Most observations were concentrated between approximately 28°C and 34°C, with relatively sparse observations above the 90^th^ percentile, indicating limited empirical support at the upper temperature tail.


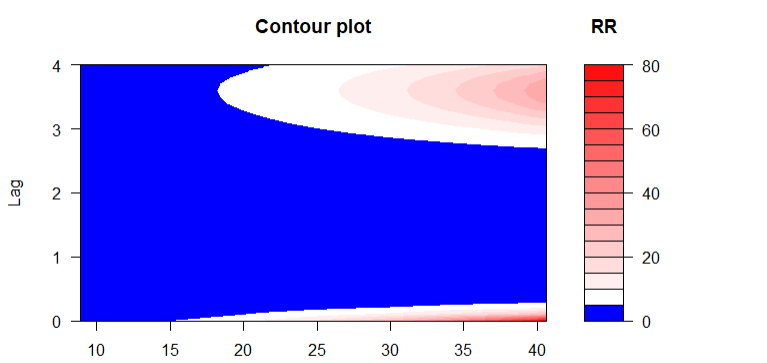


Suppl Figure 3: Contour plot of lag–temperature–risk relationship for rotavirus infection (DLNM model)


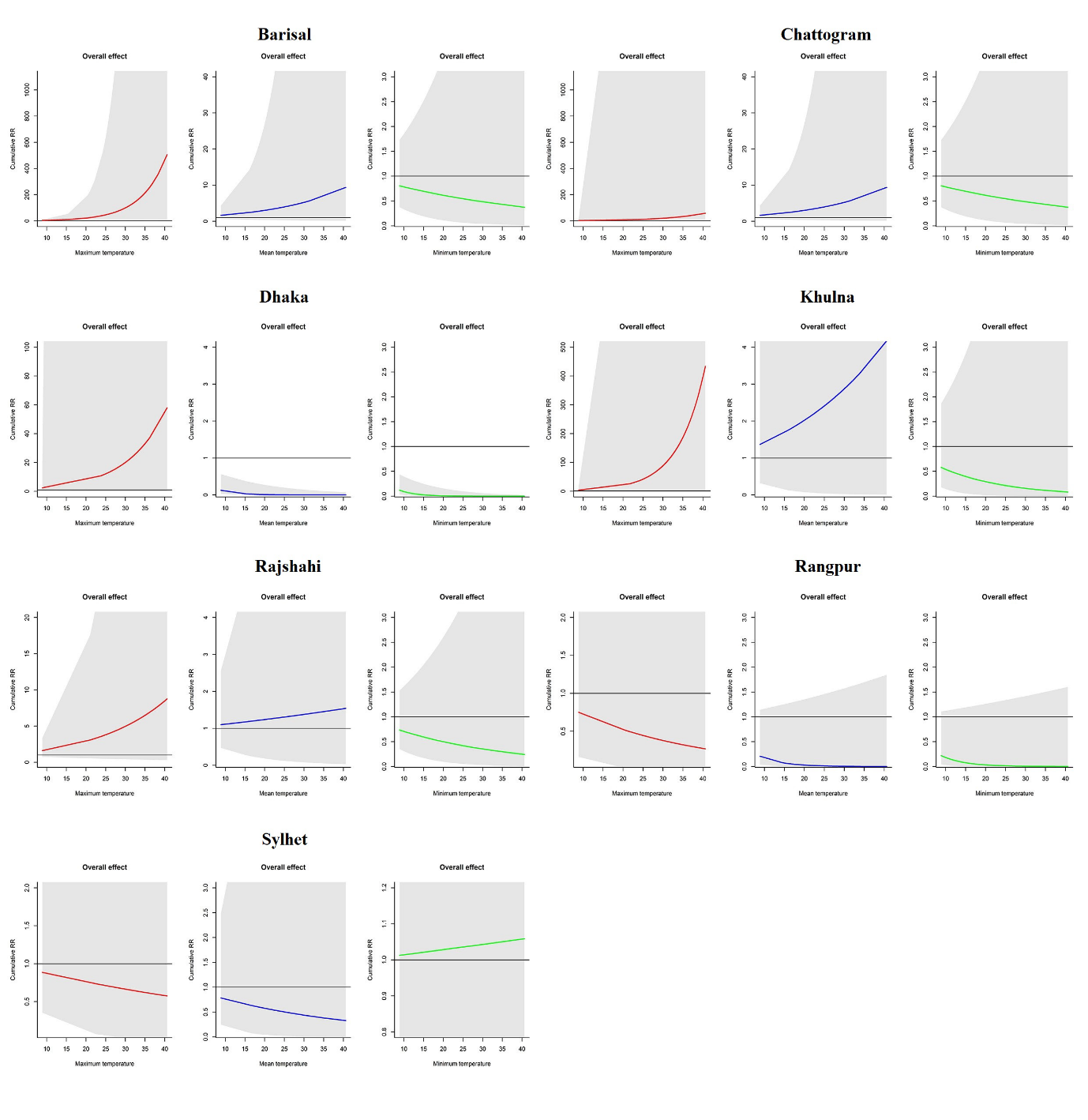


Suppl Figure 4: Overall cumulative plot of risk at different temperatures per administrative division level. All cumulative relative risks (RRs) are expressed relative to the division-specific reference temperature, defined as the average weekly mean temperature for each division during the study period (Barisal: 25.6 °C; Chattogram: 26.2 °C; Dhaka: 24.9 °C; Khulna: 25.9 °C; Rajshahi: 25.3 °C; Rangpur: 24.7 °C; Sylhet: 24.9 °C).

Suppl Table 2: Sensitivity analysis of attributable number and attributable fraction (AF) using alternative reference temperatures

| **Administrative level** | **Baseline** | **Reference temperature (°C)** | **Total attributable number** | **Total attributable fraction (%)** |
| --- | --- | --- | --- | --- |
| Overall (countrywide) | Median t-max | 31.6 | −3,038 | −70.1 |
|  | MRT | 20.5 | 3,237 | 74.6 |
| Division | | | | |
| Barisal | Median t-max | 31.7 | −267 | −27.7 |
|  | MRT | 22.5 | 666 | 68.9 |
| Chattogram | Median t-max | 30.9 | −57 | −23.6 |
|  | MRT | 23.8 | 96 | 39.3 |
| Dhaka | Median t-max | 30.9 | 76 | 11.7 |
|  | MRT | 36.5 | 257 | 39.7 |
| Khulna | Median t-max | 32.8 | −331 | −95.2 |
|  | MRT | 22.0 | 212 | 61.1 |
| Rajshahi | Median t-max | 32.9 | −348 | −29.4 |
|  | MRT | 21.5 | 350 | 29.5 |
| Rangpur | Median t-max | 30.9 | 43 | 15.1 |
|  | MRT | 36.5 | 83 | 29.2 |
| Sylhet | Median t-max | 31.3 | 5 | 0.8 |
|  | MRT | 38.0 | 63 | 9.4 |

** MRT: Minimum-risk temperature - corresponds to the temperature associated with the lowest cumulative relative risk in the DLNM; Negative attributable fractions indicate that the specified reference temperature lies on a higher-risk portion of the exposure–response curve relative to the observed temperature distribution. Minimum-risk temperature (MRT) corresponds to the temperature associated with the lowest cumulative relative risk in the DLNM.*


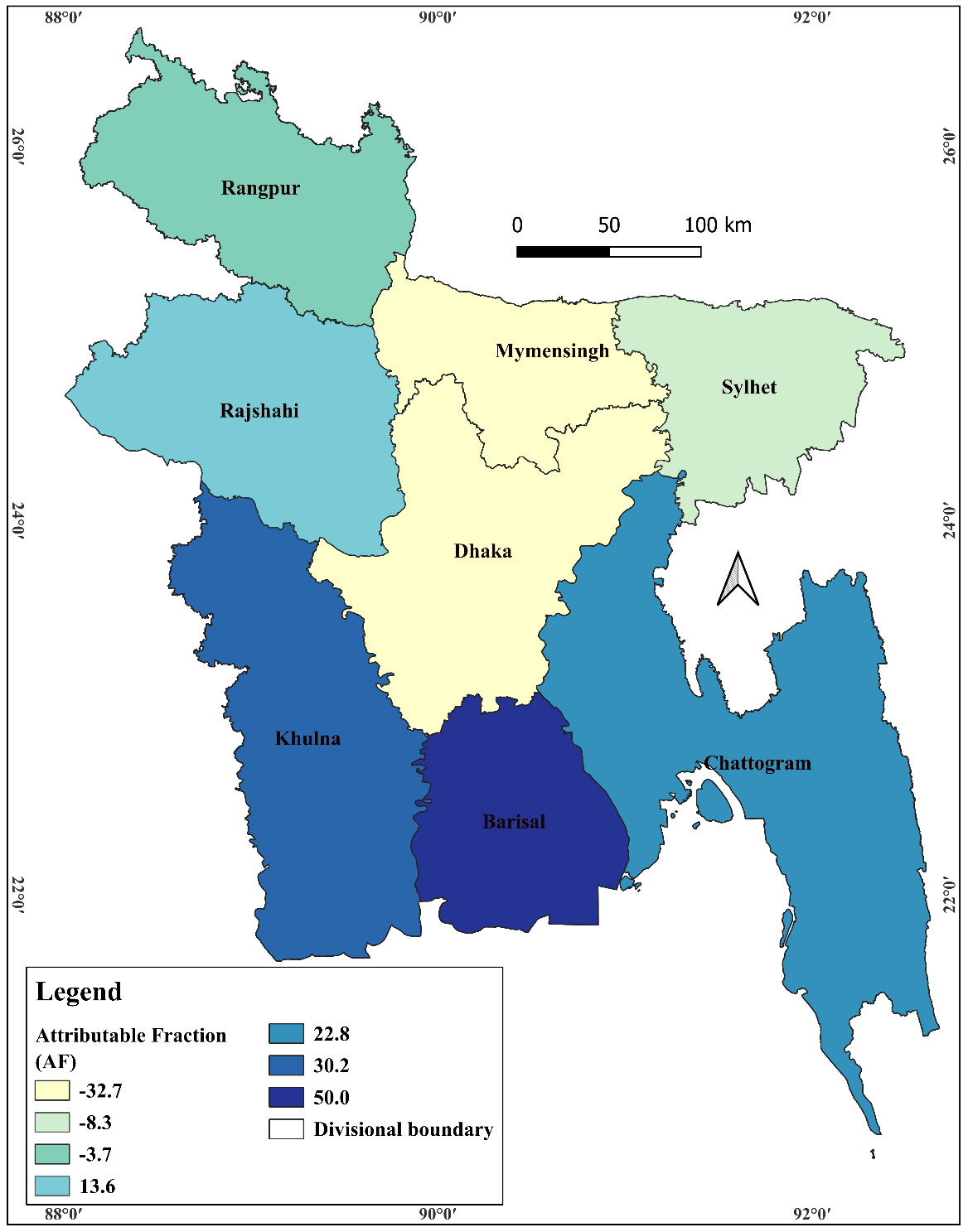


Suppl Figure 5: Map of the seven surveillance divisions included in the study, provided to visually contextualise the regional heterogeneity observed in division-specific attributable fractions and temperature–rotavirus associations. Mymensingh, established as a separate division in 2015, was retained within the Dhaka division analytical category throughout the study period for consistency.
